# Using high-frequency monitoring data to quantify city-wide suspended-sediment load and evaluate TMDL goals

**DOI:** 10.1007/s10661-023-11905-3

**Published:** 2023-10-26

**Authors:** Samuel A. Miller, James S. Webber, John D. Jastram, Marcus F. Aguilar

**Affiliations:** 1 U.S. Geological Survey, Virginia and West Virginia Water Science Center, 1730 East Parham Road, Richmond, VA 23228 USA; 2 City of Roanoke, Department of Public Works, 1802 Courtland Rd. NE, Roanoke, VA 24012 USA

**Keywords:** Suspended sediment, Turbidity, TMDL, Water quality, Urban hydrology, Surrogate regression model

## Abstract

Excess sediment is a common reason water bodies in the USA become listed as impaired resulting in total maximum daily loads (TMDL) that require municipalities to invest millions of dollars annually on management practices aimed at reducing suspended-sediment loads (SSLs), yet monitoring data are rarely used to quantify SSLs and track TMDL progress. A monitoring network was created to quantify the SSL from the City of Roanoke, Virginia, USA (CoR), to the Roanoke River and Tinker Creek and help guide TMDL assessment and implementation. Suspended-sediment concentrations were estimated between 2020 and 2022 from high-frequency turbidity data using surrogate linear-regression models. Sixty-one percent of the total three-year SSL resulted from five large storm events. The average suspended-sediment yield from the CoR (58.1 metric tons/km^2^/year) was similar to other urban watersheds in the Eastern United States; however, the yield was nearly five times larger than the TMDL allocation (12.2 metric tons/km^2^/year). The TMDL allocated load was modeled based on a predominantly forested reference watershed and may not be a practical target for highly impervious watersheds within the CoR. The TMDL model used daily input data which likely does not capture the full range of SSLs during storm events, particularly from flashy urban streams. The average SSL following the five large storm events doubled that of the CoR’s annual allocated load from the TMDL. The results of this study highlight the importance of using high-frequency monitoring data to accurately estimate SSLs and evaluate TMDLs in urban areas.

**Supplementary Information:**

The online version contains supplementary material available at 10.1007/s10661-023-11905-3.

## Introduction

Although the balanced natural process of sediment transport is important for landscape formation and river dynamics, human activities commonly disrupt this balance, resulting in excess sediment transport that can have lingering impacts lasting decades to centuries (Pizzuto et al., 2017). Anthropogenic factors increased sediment delivery by 215% from 1950 to 2010 and now dominate global sediment fluxes (Syvitski et al., 2022). Excess suspended sediment can have direct impacts to biota, such as reduced light penetration for photosynthesis and burial of streambed habitat, indirect effects associated with contaminant and nutrient transport, and is considered one of the leading causes of stream-habitat decline (Davies-Colley & Smith, 2007; Noe et al., 2020; Wood & Armitage, 1997). Approximately 224,000 km of rivers and streams in the USA are impacted by excess sediment which is one of the most common reasons for total maximum daily load (TMDL) development (U.S. Environmental Protection Agency, 2017), yet many TMDL implementation plans lack the high-frequency monitoring data necessary to accurately quantify pollutant loads and assess the impact of management practices on water-quality changes.

Most TMDL implementation plans rely on empirical models that use daily input data to estimate pollutant loads contributed from land sources and relatively few studies have compared modeled loads with loads calculated from in-stream monitoring data (Grismer, 2013). Although the TMDL process has been largely a success at reducing traditional point source pollution (i.e., permitted dischargers), questions remain on the impact of TMDLs at reducing diffuse sources of pollution, such as stormwater runoff from agricultural and developed land surfaces, which is the leading cause of impairment for most water bodies (United States Government Accountability Office, 2013). Unlike point source pollutants that are regulated through permitting and discharge limits, abatement of non-point source loads is largely through voluntary means since the U.S. Environmental Protection Agency (EPA) does not have authority to compel landowners to take actions to reduce such pollution (United States Government Accountability Office, 2013). In a survey of 191 established TMDLs, 83% achieved point source pollution targets, but only 20% achieved non-point source pollution targets (United States Government Accountability Office, 2013), which highlights the utility for an adaptive implementation approach in which monitoring data are used to assess progress toward attaining water quality standards and to refine implementation planning, if needed. Additionally, monitoring data are needed to evaluate the effectiveness of stormwater management projects and can help municipalities effectively invest in strategies that result in the greatest water-quality improvements. Municipalities invest millions of dollars annually in management practices aimed to reduce pollutant loads, yet funding to establish baseline pollutant loads and track the water quality changes associated with TMDL implementation are often inadequate (United States Government Accountability Office, 2013). One challenge in TMDL implementation is accurately assigning pollutant loads to permitted localities since watershed and political boundaries do not typically overlap; therefore, accurate estimates of pollutant loads across city boundaries are often unavailable. In this study, we demonstrate how a more robust monitoring design can be used to quantify pollutant loads from a mid-size city.

Developed areas can be especially prone to excess sediment erosion due to impervious surfaces that result in greater amounts of stormwater runoff, flashier streamflow, and altered channel morphology. As a result, the natural balance between sediment supply and deposition can be disrupted, leading to progressive channel erosion and enlargement with increased sediment carrying capacity (Walsh et al., 2005; Wolman, 1967). Numerous studies from urban and suburban watersheds in humid-temperate climates have identified streambank erosion, accelerated by peak streamflow, as the dominant source of suspended sediment (Allmendinger et al., 2007; Carter et al., 2003; Cashman et al., 2018; Devereux et al., 2010; Gellis et al., 2017; Smith & Wilcock, 2015; Trimble, 1997). The greatest upland sources of sediment (i.e., non-channel) in developed areas include active construction sites and gravel sources (Russell et al., 2019). Although impervious surfaces have been shown to generate relatively lower sediment yields due to reduced supply, sediment transport capacity increases substantially due to greater drainage connectivity (Russell et al., 2019). These cumulative effects can lead to increased floodplain deposition due to more frequent flood events caused by increased peak streamflow (Hupp et al., 2013). Previous research indicates that as the proportion of impervious surfaces increases, peak streamflow rates rise exponentially (Corbett et al., 1997), and even low levels of urbanization (2–10% imperviousness) can cause geomorphic change (Vietz et al., 2016). For these reasons, many studies have reported greater suspended-sediment yield (SSY) from urban drainage networks compared to background conditions, especially in humid-temperate climates similar to the City of Roanoke (CoR, see for example, Russell et al., 2017).

Excess sediment was identified by the Virginia Department of Environmental Quality (DEQ) as the most probable stressor impairing benthic macroinvertebrate communities along sections of the Roanoke River near the CoR (Virginia Department of Environmental Quality, 2006, 2016). As part of the Implementation Plan following the TMDL, the CoR was required to implement suspended-sediment control measures within the City’s service area. Results from bioassessments identified urban stormwater runoff, streambank erosion, and sediment loss as potential sources of benthic macroinvertebrate habitat impairment (Virginia Department of Environmental Quality, 2016). The DEQ established a numeric TMDL endpoint for the Roanoke River based on the sediment-loading rate in a non-impaired, reference watershed. Sediment loading from instream erosion and all land-use sources except forest required a reduction of 75% from a modeled preexisting sediment load to meet the TMDL endpoint. Since the CoR contains about 10% forest land, an overall 67.5% reduction in sediment loading would be required. The CoR has begun implementing management strategies to comply with the TMDL on waterways within its jurisdiction; however, the feasibility and ecological benefit of reducing suspended-sediment loads by 67.5% across the entire CoR service area and the accuracy in the sediment loading rates estimated by the TMDL are unknown. Accurate quantification of suspended-sediment loads (SSLs) is necessary to assess baseline conditions, evaluate progress towards meeting TMDL goals, and account for contributions from the CoR.

The objective of this paper is to evaluate the SSL contributed from the CoR to its two major waterways: the Roanoke River and Tinker Creek. This overall objective can help guide sediment-reducing activities within the CoR and for other large municipalities. This study uses high-frequency monitoring data to better understand the hydrologic conditions that generate SSL and to evaluate load reductions required by the TMDL. This paper introduces a novel monitoring design and analytical approach to estimate SSL at sub-daily timesteps within a city boundary. For many cities, accurate monitoring-based estimates of SSL remain a key gap in TMDL implementation and assessment. Specifically, the objectives of this paper are to (1) describe the study design for quantifying the SSL contributed from the CoR to its major waterways, (2) estimate and characterize the SSL from five sediment-monitoring stations during water years (October 1–September 30; “years”) 2020 through 2022, and (3) contextualize the results by comparing the SSY from the CoR to the TMDL allocated SSY and nearby urban watersheds in the Eastern United States.

## Material and methods

### Study area

**Fig. 1:**
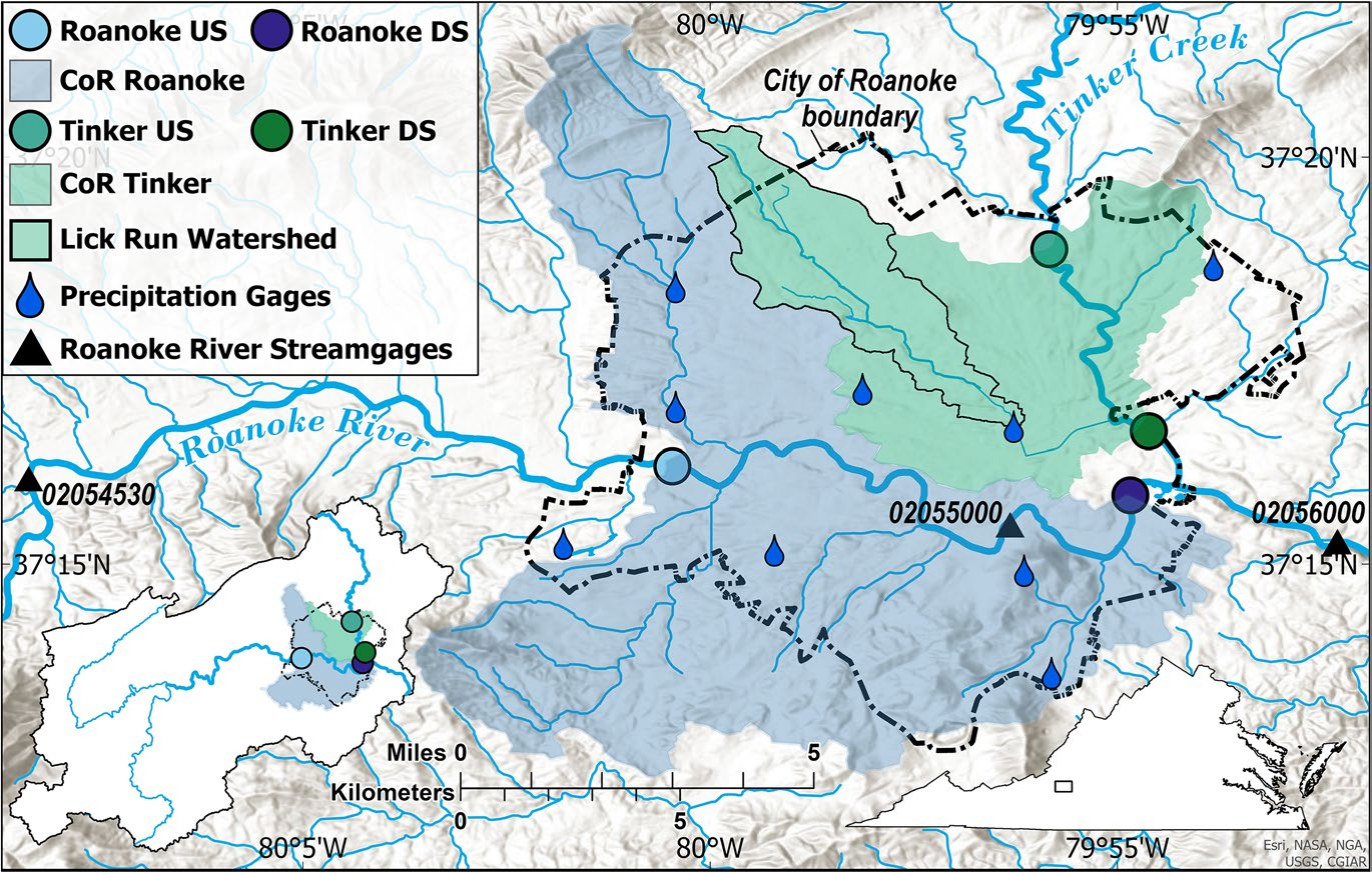
Study area with the locations of the upstream (US) and downstream (DS) sediment*-*monitoring stations on the Roanoke River and Tinker Creek, the Lick Run study watershed, and precipitation and Roanoke River streamgages and USGS site numbers. The blue and green shaded areas represent the City of Roanoke’s suspended-sediment contribution to the Roanoke River (CoR Roanoke) and Tinker Creek (CoR Tinker), which combined are referred to as the suspended-sediment contribution area (CoR SCA). Two inset maps provide the location of the study area within the Roanoke DS and Tinker DS watersheds (bottom left) and the Commonwealth of Virginia (bottom right)

The CoR (111 km^2^) is the largest municipality in southwest Virginia with a population just over 100,000 (U. S. Census Bureau, 2020) and is located within both the Blue Ridge and Valley and Ridge Physiographic Provinces (Fenneman, 1946) (Fig. 1; Table 1). In 2019, developed area within the CoR occupied 87% of the land, of which 42% was impervious, 10% of the city was forested, and small remaining portions were open fields (Dewitz & U.S. Geological Survey, 2021). Because the city’s business district developed in downtown Roanoke during the late 1800s, perennial streams were buried and many surface streambeds were channelized and lined with concrete to increase stormwater capacity (Aguilar et al., 2019). As a result, the natural hydrography and sediment cycle within the CoR has been substantially modified and the central business district and surrounding developed areas frequently experience flash flooding (Brendel et al., 2021). Within the CoR, the valley slopes adjacent to the main channels can exceed 40% in certain locations, which requires maintained vegetation to mitigate excessive streambank erosion (Dymond et al., 2017).

**Table 1:** Drainage areas and dominant National Land Cover Database (NLCD) (Dewitz & U.S. Geological Survey, 2021) categories for the watersheds draining to the sediment-monitoring stations, the contribution areas from the CoR, and the CoR administrative boundary. The first five rows are monitored U.S. Geological Survey (USGS) streamgages; CoR Roanoke, contribution from the City of Roanoke to the Roanoke River; CoR Tinker Creek, contribution from the City of Roanoke to Tinker Creek; CoR SCA, total City of Roanoke suspended-sediment contribution area; CoR Admin, City of Roanoke Administrative boundary

		Drainage area	NLCD Category (%)		
Name	USGS Site Number	(km^2^)	Impervious	Developed	Forest
Lick Run	0205551460	13.1	47	95	3
Roanoke US	02054750	911	5	14	73
Roanoke DS	02055080	1010	7	20	68
Tinker US	02055379	162	11	28	53
Tinker DS	0205551614	202	18	40	44
CoR Roanoke		98.4	32	75	22
CoR Tinker		39.9	48	89	6
CoR SCA		139	36	79	18
CoR Admin		111	42	87	10

A monitoring network was established to quantify SSLs delivered from the CoR to two major waterways: the Roanoke River and Tinker Creek. Sediment-monitoring stations were operated at the approximate locations where these two waterways enter and exit the CoR boundary. The upstream SSL was subtracted from the downstream SSL to determine the SSL contributed from the CoR to these waterways denoted by the shaded areas in Fig. 1 and referred to as the CoR suspended-sediment contribution area (CoR SCA). Upstream and downstream stations are denoted with “US” and “DS,” respectively. The contributions to the Roanoke River and Tinker Creek from the CoR are denoted as “CoR Roanoke” (98.4 km^2^) and “CoR Tinker” (39.9 km^2^), respectively. In addition to the four sediment-monitoring stations located on the Roanoke River and Tinker Creek, a fifth station is located on Lick Run, a small urban watershed (13.1 km^2^) nested within the Tinker DS study watershed and almost completely contained within the CoR boundary (Fig. 1). The Lick Run monitoring station has a longer history of data collection and is more representative of the CoR in terms of land cover and imperviousness compared to the stations on the Roanoke River and Tinker Creek that drain much larger areas upstream of the city (Table 1; Table 6 and Table 7 in the Appendix).

The CoR SCA (139 km^2^) is slightly larger than the CoR administrative boundary (111 km^2^) but has similar land-cover characteristics, geology, and soils (Table 1; Table 6 and Table 7 in the Appendix). The geology underlying the CoR SCA is composed primarily of clastic sedimentary rocks containing shale and siltstone, and carbonate rocks, including dolostone and limestone (Dicken et al., 2005). Soils within the CoR SCA typically are composed of clay loam, silty clay loam, or sandy clay and are most commonly categorized as hydrologic soil group “D,” which has very low infiltration rates and the highest runoff potential (Soil Survey Staff, 2020).

Watershed drainage areas for the Tinker Creek sediment-monitoring stations are much smaller compared to the Roanoke River sediment-monitoring stations (Table 1). Forest is the dominant land-cover class for both Roanoke River and Tinker Creek watersheds. Impervious surfaces occupy a greater percentage of area in the Tinker DS watershed compared to the Roanoke DS watershed, because the CoR occupies a greater percentage of area in the Tinker DS watershed (20%) compared to the Roanoke DS watershed (10%). Clastic sedimentary rocks, primarily shale and siltstone, are the dominant lithologies for the Roanoke River and Tinker Creek watersheds (Dicken et al., 2005) (Table 6 in the Appendix). However, carbonate lithologies composed of dolostone and limestone occupy a larger percentage of land in the Tinker Creek watersheds and are common in the river valleys. The dominant hydrologic soil group for the Roanoke River and Tinker Creek watersheds is classified as “B,” soils consisting of fine to moderately coarse textures with moderate infiltration rates (Soil Survey Staff, 2020) (Table 7 in the Appendix).

### Precipitation monitoring network and characterization

Nine weighing-type precipitation gages were installed within the CoR (U.S. Geological Survey [USGS] site numbers: 0205551460, 371339079554400, 371459079560300, 371518079591700, 371520080015100, 371657080002800, 371709079580800, 371824080002600, 371840079534900; Fig. 1). Data were collected at 5-min intervals in accordance with published USGS standards (U.S. Geological Survey, 2015) and are available from the National Water Information System database (U.S. Geological Survey, 2023) and in ESM1. ESM4 contains a data dictionary for all supplementary material. Mean 5-min precipitation data from the nine gages were calculated and aggregated annually. An algorithm was developed to identify individual precipitation events using an 8-h minimum inter-event period (Hopkins et al., 2020; Miller et al., 2022). Events with at least 0.25 cm (0.1 in) of cumulative precipitation were summarized by total precipitation amount, maximum 1-h intensity, and duration to determine the effects of event characteristics on SSL transport. Mean annual event characteristics among the nine gages were evaluated for significant differences using Tukey’s Honest Significant Difference test to compare precipitation patterns between the three years. All analyses were conducted in R version 4.1.0. (R Core Team, 2021) and significant differences were determined when *p* < 0.05 unless stated otherwise. Annual precipitation data were compared to the long-term average reported from the xmACIS RNK forecast area, “Roanoke Area” station (https://xmacis.rcc-acis.org). The areal extent of the nine USGS precipitation gages is similar to the stations used to report the long-term average.

### Streamflow measurement and estimation

Continuous streamflow data were monitored at the two Tinker Creek monitoring stations and at Lick Run with stage-discharge rating curves following standard USGS methods (Rantz, 1982; Sauer & Turnipseed, 2010). Continuous streamflow data were estimated at the two Roanoke River monitoring stations using watershed area ratios to scale streamflow from nearby streamgages (Gianfagna et al., 2015; Jastram et al., 2015). Streamflow for Roanoke US was estimated from streamflow collected at USGS site numbers 02054530 and 02055000, whereas streamflow at Roanoke DS was estimated from USGS site numbers 02055000 and 02056000 (Fig. 1) (U.S. Geological Survey, 2023). Streamflow was estimated using the drainage area and streamflow from the upstream and downstream gages:

1 $${Q}_{ungaged}={A}_{ungaged}\times \frac{\left({Q}_{upstream}+{Q}_{downstream}\right)}{\left({A}_{upstream}+{A}_{downstream}\right)}$$

where *Q* represents streamflow and *A* represents watershed area. This estimation technique assumes that as watershed area increases, streamflow rate increases at a fixed rate per unit area; however, the streamflow rate per unit area is likely to increase along this section of the Roanoke River given the increase in developed land cover from Roanoke US to Roanoke DS (Table 1). Therefore, streamflow is likely overestimated using Eq. 1 since the downstream gaging stations are more developed than the ungaged sediment monitoring stations. To resolve this potential overestimation, we also estimated the area-scaled streamflow at Roanoke US and Roanoke DS using only one gaged location in between Roanoke US and Roanoke DS (USGS site number 02055000) which underestimated streamflow within CoR Roanoke since the streamflow per unit area was fixed to one location:

2 $${Q}_{ungaged}={A}_{ungaged}\times \frac{\left({Q}_{02055000}\right)}{\left({A}_{02055000}\right)}$$

Streamflow estimated from Eqs. 1 and 2 likely reflect the maximum and minimum scenarios, respectively; therefore, the streamflow for Roanoke US and Roanoke DS was computed as the average of Eq. 1 and Eq. 2 and available in ESM3.

Annual streamflow contributions from the CoR Roanoke and CoR Tinker were calculated by subtracting the streamflow measured at the upstream stations from the downstream stations. Total streamflow from the CoR SCA was calculated by adding the streamflow from CoR Roanoke and CoR Tinker. Streamflow volume was converted to streamflow yield by dividing by watershed area to make comparisons among watersheds and contributing areas with different drainage areas.

### Suspended-sediment concentration estimation using surrogate regression models

Manual suspended-sediment concentration (SSC) samples (*n* = 519) were collected under varying hydrologic conditions to characterize suspended-sediment transport (ESM2). Quality assurance samples consisting of field blanks (*n*=14) and replicate samples (*n*=40) were collected to evaluate SSC results. The hydrologic and water quality conditions for each sample and the collection equipment and method identifiers are included in the supplemental calibration data. Measured SSCs were compared to continuous water-quality data to support the computation of continuous SSL using surrogate linear regression models (Guy, 1969). All five stations were equipped with in situ sensors to measure water temperature, specific conductance, dissolved oxygen, pH, and turbidity at 15-min intervals in accordance with published USGS standards (U.S. Geological Survey, 2015; Wagner et al., 2006). Sensors were installed approximately mid-stream and cleaned and inspected for instrument drift every other month. The primary water-quality parameter was turbidity, which has been used in surrogate regression models to estimate SSC along the Roanoke River within the CoR (Jastram et al., 2010; Jastram et al., 2015) and neighboring regions (Aulenbach et al., 2017; Aulenbach et al., 2022; Jastram, 2014; Jastram et al., 2009; Porter, 2022; Porter et al., 2020; Williamson & Crawford, 2011). Turbidity represents the optical properties of a water sample that cause light rays to be scattered and absorbed; thus, the presence of suspended material such as clay, silt, and fine organic matter cause turbid water; the magnitude of turbidity often is proportional to SSC (Rasmussen et al., 2009).

In addition to turbidity, the remaining water-quality parameters, streamflow, and seasonal terms were considered explanatory variables in SSC surrogate regression models. For the four sediment-monitoring stations located on the Roanoke River and Tinker Creek, evaluation of whether the turbidity-SSC relation varied between upstream and downstream locations was accomplished by adding a term to determine significance of location within a pooled model calibrated using data from both stations (Helsel et al., 2020; Jastram et al., 2015). The final models were selected using a best subsets approach based on numerous criteria, including residual standard error (RSE), adjusted coefficient of determination (R^2^_a_), Akaike information criterion, predicted residual error sum of squares, variance inflation factor, and inspection of model residuals (Helsel et al., 2020). Turbidity, streamflow, and SSC were log-transformed to make model residuals more symmetric, linear, and homoscedastic, so that assumptions for linear regression were met. Model estimates were retransformed to original units using a nonparametric bias correction factor (Helsel et al., 2020). Surrogate linear regression models and associated 95% confidence intervals were generated using the package “stats” (R Core Team, 2021).

Suspended-sediment concentrations in 48 samples collected during low turbidity conditions (<10 FNU) showed unreasonably high SSC (up to 100 milligrams per liter [mg/L]) and were omitted from surrogate linear regression models but are included in ESM2 for reference. These “high-bias” samples were selected by having turbidity less than 10 FNU and a ratio of SSC to turbidity greater than five. Recent samples collected since water year 2022 suggest that SSCs from the omitted samples were possibly inaccurate because of precipitation from dissolved material. After removing the high-bias samples, four outlier data points from Lick Run were identified using the Bonferroni Outlier Test and removed using the R package “car” (Fox & Weisberg, 2019). An autosampler is used to collect storm samples at Lick Run since streamflow responds promptly to rain events in this smaller, highly impervious watershed and the four identified outlier data points were likely caused by unrepresentative sampling during the flashy storm events. The autosampler intake was installed mid-stream at mid-depth in 2016 and collects samples when turbidity and rate-of-change stage thresholds are exceeded. Samples were purged prior to collection and collected within a week.

When turbidity data were unavailable due to sensor fouling, estimation techniques were used to fill in missing turbidity values. Relations between the upstream and downstream turbidity sensors were calculated using linear regression to fill in missing turbidity data at the Roanoke River and Tinker Creek monitoring stations. Downstream missing turbidity data were estimated from the relation with upstream turbidity data, and vice versa. Cross-correlation functions were calculated for the two pairs of monitoring stations to determine and apply the lag time in turbidity sensor data when filling missing data using the R package “stats” (R Core Team, 2021). Following these steps, linear interpolation was used to fill short periods of missing data (< 4 h) and longer periods of missing data (up to 5 days) that occurred during low streamflow conditions when turbidity was typically stable. Both original and interpolated turbidity data are available in ESM3. For remaining periods of missing turbidity data, SSC was estimated from streamflow surrogate regression models that did not include turbidity data (Robertson et al., 2018).

### Suspended-sediment load estimation

Instantaneous SSCs obtained from surrogate regression models were multiplied by streamflow to estimate SSL at 15-min intervals from the five sediment-monitoring stations and annually aggregated. The SSY was calculated by dividing SSL by watershed area to normalize SSLs among watersheds with different drainage areas. The SSL from Roanoke US was subtracted from the SSL from Roanoke DS to determine the SSL from the CoR to the Roanoke River (CoR Roanoke). The SSY from CoR Roanoke was calculated by dividing SSL by the area difference between the two watersheds (98.4 km^2^). Likewise, the SSL from Tinker US was subtracted from the SSL from Tinker DS to determine the SSL and SSY from the CoR to Tinker Creek (CoR Tinker ~ 39.9 km^2^). The total SSL and SSY from the CoR to the Roanoke River and Tinker Creek (CoR SCA) was calculated by adding the SSL from CoR Roanoke and CoR Tinker and dividing by the CoR SCA (139 km^2^). Annual SSY was compared to the TMDL allocation and SSY calculated from other regional urban watersheds to contextualize the suspended-sediment transport estimated in this study.

In addition to annual totals, streamflow and SSLs were aggregated for individual precipitation events. The minimum inter-event period for detecting precipitation events outlined above occasionally resulted in successive storms that produced one distinct hydrograph response. Storms that began within 24 h of the end of the previous event were combined into one event and precipitation metrics were summed with those of the successive events because the hydrologic response was impacted by both events (Hopkins et al., 2020). Total SSL from the CoR SCA was calculated for each precipitation event from initiation to 2 days after the event ended and compared to precipitation event characteristics. This approach provided a uniform method to compare the SSL response to precipitation events that generally captured the rising and falling limbs of the hydrograph. All instantaneous streamflow, water quality, SSC, and SSL data are available in ESM3.

## Results and discussion

### Precipitation and streamflow

**Fig. 2:**
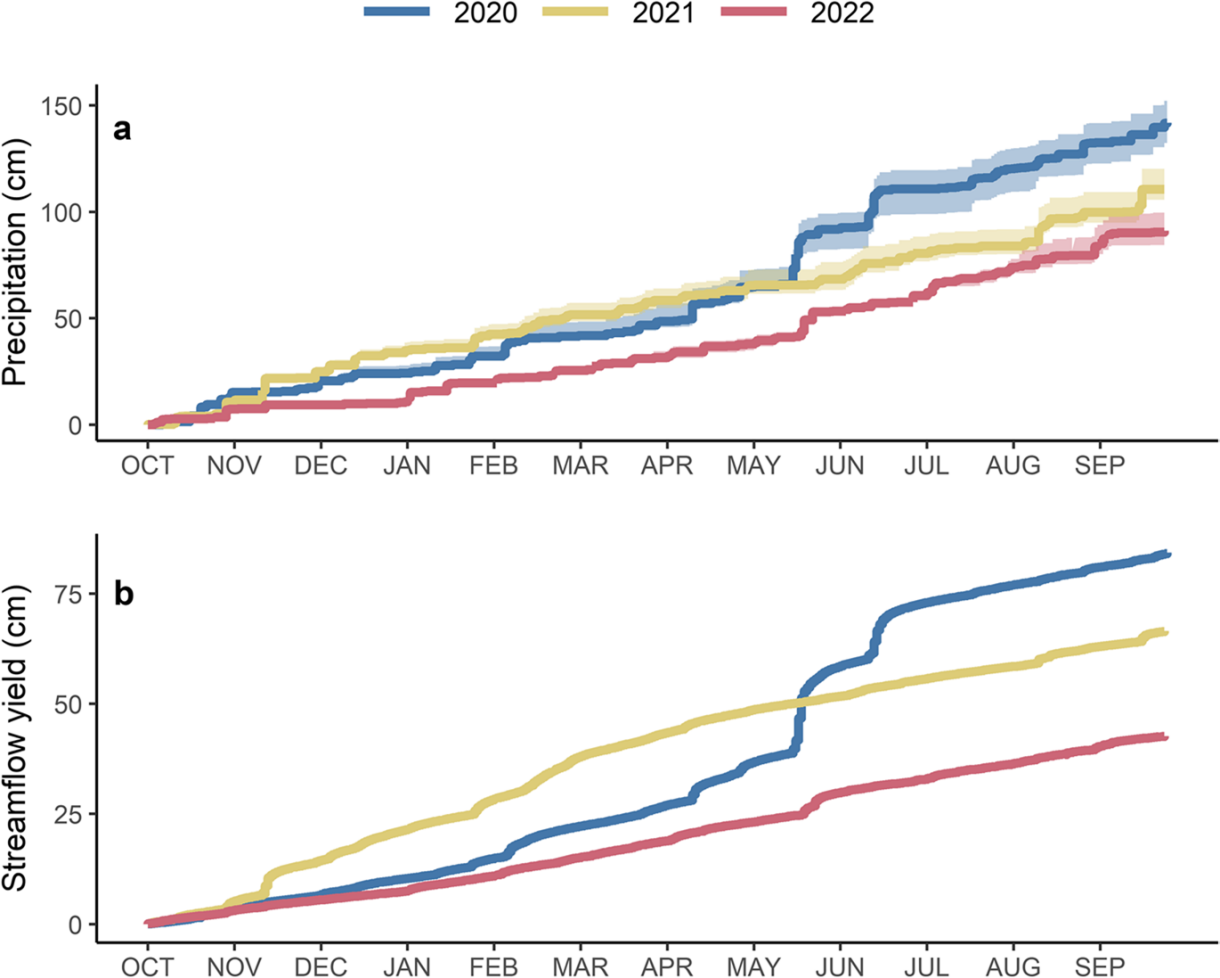
Cumulative precipitation during 2020 (blue), 2021 (yellow), and 2022 (red) (**a**). The bold lines represent mean precipitation from the nine precipitation gages; the transparent background represents the range from individual stations. Cumulative streamflow yield from the City of Roanoke sediment contribution area (CoR SCA) during 2020 (blue), 2021 (yellow), and 2022 (red) (**b**)

Precipitation recorded in 2020, 2021, and 2022 represented above average, average, and below average conditions compared to the long-term average from 1913 to 2021 for the Roanoke area (106 cm), respectively (Fig. 2a; Table 2). Total precipitation was significantly different in all 3 years and there were significantly more precipitation events that generated at least 0.25 cm (0.1 in) in 2020 compared to 2021 or 2022. In general, mean event precipitation amounts, duration, and maximum 1-h intensity were significantly greater in 2020 compared to 2021 and 2022. Overall, these 0.25-cm events contributed to about 97% of the total precipitation during the study period. Precipitation accumulation was relatively steady during the first half of the three water years, but large storm events in the spring and summer months resulted in abrupt increases in cumulative precipitation.

**Table 2:** Mean ± standard deviation for precipitation metrics from the nine gages during the study period summarized by water year including total precipitation (P), number of events with greater than 0.25 cm, mean event precipitation amount, duration, and mean maximum 1-h intensity from events. Unique letters in parentheses indicate significant differences among water years according to Tukey’s test

Water year	Total P (cm)	Events (*n*)	Event P (cm)	Event duration (hr)	Event max intensity (cm/hr)
2020	142 *±* 6 (a)	69 *±* 2 (a)	2.0 *±* 0.1 (a)	10.0 *±* 0.9 (a)	0.75 *±* 0.04 (a)
2021	110 *±* 4 (b)	63 *±* 2 (b)	1.7 *±* 0.1 (b)	8.9 *±* 0.4 (b)	0.68 *±* 0.05 (b)
2022	91 *±* 4 (c)	60 *±* 4 (b)	1.4 *±* 0.1 (c)	7.1 *±* 0.5 (c)	0.71 *±* 0.05 (ab)

There were 40 storm events that generated at least 2.54 cm (1 in) of precipitation, 16 of which occurred in 2020. These larger events were responsible for over half of the precipitation recorded (51%) during the 3-year study period. Five of the largest precipitation events were responsible for three-fifths of the SSL transported from the CoR SCA and are examined in further detail in the following sections (Table 3). These events had an average of 12.4 cm (4.9 in) of precipitation, or 18% of total precipitation recorded during the study period, generally occurred over multiple days, and had much greater maximum one-hour intensities compared to other precipitation events.

**Table 3:** Precipitation, streamflow yield (Q), and suspended-sediment load (SSL; metric tons) and suspended-sediment yield (SSY) characteristics for the five storm events that generated the largest SSL, mean and total statistics from the five events, and mean statistics from all 0.25-cm and 2.5-cm storms. Streamflow yield, SSL, and SSY are from the City of Roanoke sediment contribution area (CoR SCA) and the contributions from the CoR to the Roanoke River (CoR Roanoke) and Tinker Creek (CoR Tinker). Streamflow yield, SSL, and SSY were summed from the moment the precipitation event began through two days after the precipitation event ended. Event duration does not include the additional two-day period

			CoR SCA			CoR Roanoke		CoR Tinker	
Start Date	Duration (hr)	P (cm)	Q (cm)	SSL (t)	SSY (t/km^2^/day)	Q (cm)	SSY (t/km^2^/day)	Q (cm)	SSY (t/km^2^/day)
2/5/2020	44.3	6.4	2.3	1773	7.6	2.1	8.7	2.7	5.0
4/12/2020	17.2	7.4	2.9	1779	19.7	2.5	16.0	3.9	29.0
5/18/2020	86.7	22.0	14.5	4226	9.3	13.8	9.1	16.2	9.8
6/14/2020	86.8	16.1	8.5	3468	7.6	7.9	7.4	10.1	8.1
11/10/2020	41.6	10.2	4.5	3512	16.1	4.0	19.1	5.6	8.6
Mean	55.3	12.4	6.5	2952	12.0	6.1	12.0	7.7	12.1
Total	276.6	62.1	32.7	14,758		30.4		38.5	
0.25-cm events	20.2	2.1	0.8	147	1.0	0.7	1.1	0.9	0.9
2.5-cm events	38.1	5.2	1.7	521	3.7	1.5	3.9	2.0	3.3

**Fig. 3:**
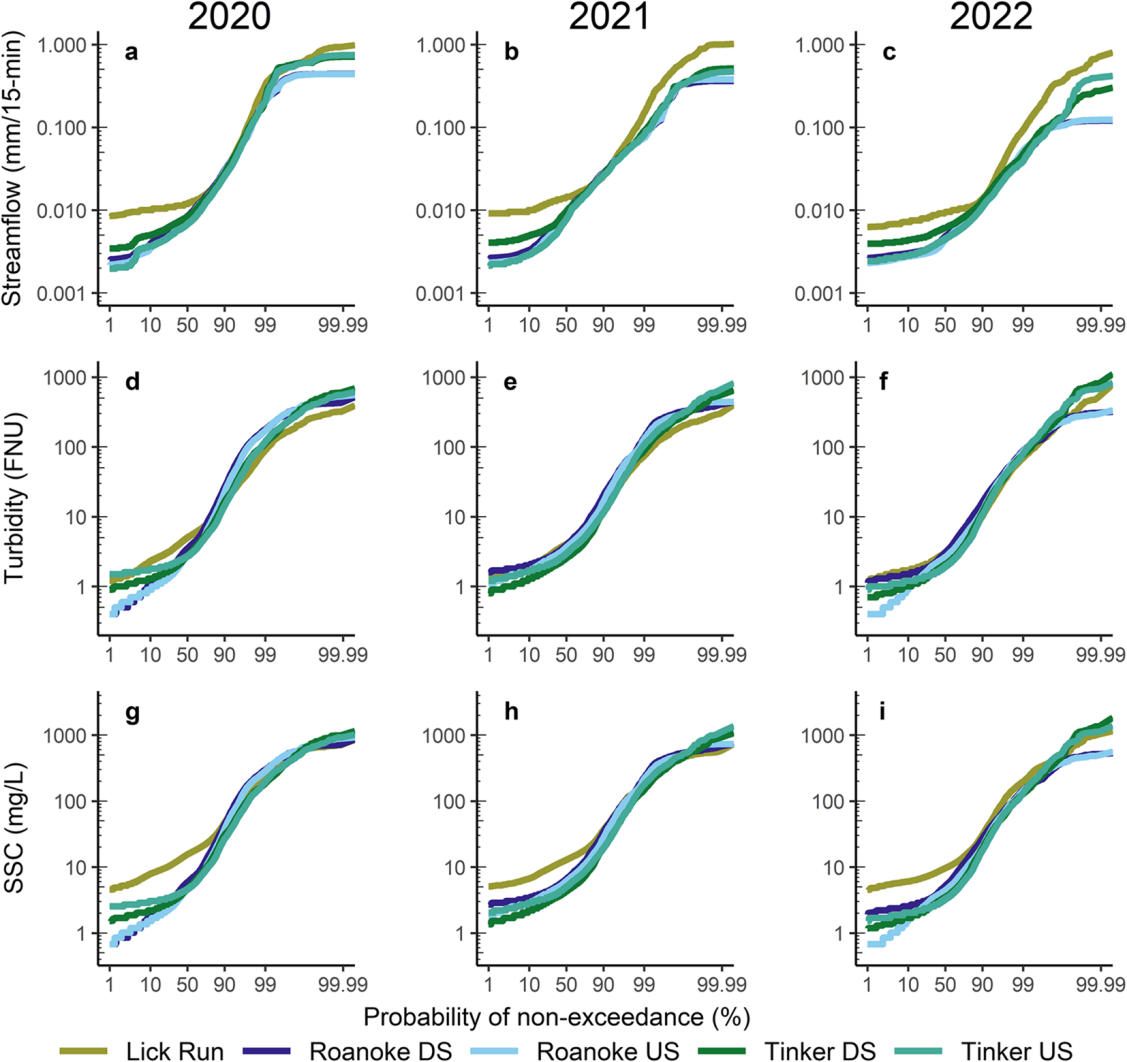
Streamflow yield (**a**, **b**, **c**), turbidity (**d**, **e**, **f**), and suspended-sediment concentration (SSC, **g**, **h**, **i**) probability of non-exceedance plots for the sediment-monitoring stations during 2020, 2021, and 2022

Streamflow was strongly influenced by precipitation and was greatest for all monitoring stations in 2020 (Fig. 2b; Table 4). Streamflow yield in the CoR SCA was twice as high in 2020 (84.4 cm) compared to 2022 (42.7 cm) despite precipitation only being about 1.5 times as great in 2020 (Table 2). In all years, the largest streamflow yields occurred in areas with greater percentages of developed land (Lick Run, CoR Roanoke, CoR Tinker, and CoR SCA) and downstream stations consistently recorded greater streamflow and streamflow yield (Table 4; Fig. 3a–c; Fig. 7 a–c in the Appendix). The streamflow responses following the five large multi-day storm events (Table 3) were responsible for 17% of the total streamflow yield from the CoR SCA over the 3-year study period yet occurred during only 2% of the period of record. During these five events, the majority of streamflow from the CoR SCA was consistently derived from the contributions to the Roanoke River (CoR Roanoke); however, streamflow yield following storm events was greater in CoR Tinker likely due to the greater proportion of impervious surfaces in this contribution area which favors more direct stormwater runoff (Table 3). Many of the 343 unique precipitation events with at least 0.25-cm occurred within 24 h of the end of the previous event and produced a single distinct hydrograph response. These events were combined into one to calculate the streamflow and SSL response, resulting in 160 combined events (Table 3). These events represented 63% of the total CoR SCA streamflow yield and occurred over 41% of the period of record. Storms with at least 2.54-cm of precipitation (*n*=40) were responsible for 34% of the CoR SCA streamflow yield yet occurred over only 13% of the period of record.

**Table 4:** Streamflow yield and suspended-sediment load for each water-quality monitoring station and calculated contribution areas from the CoR during 2020, 2021, and 2022

	Streamflow yield (cm)			Suspended-sediment load (metric tons, i.e., 1000 kg)		
Name	2020	2021	2022	2020	2021	2022
Lick Run	81.4	72.7	45.2	1090	709	497
Roanoke US	58.8	48.7	24.3	54,331	26,500	8631
Roanoke DS	60.9	50.1	25.6	64,251	32,699	10,816
CoR Roanoke	79.8	63.3	37.3	9920	6198	2184
Tinker US	59.0	46.8	23.7	8189	3884	2464
Tinker DS	66.3	52.3	30.1	12,139	5024	3178
CoR Tinker	95.7	74.5	56.0	3950	1140	714
CoR SCA	**84.4**	**66.5**	**42.7**	**13,870**	**7338**	**2898**

The CoR contributed about 13% and 29% of the streamflow to Roanoke DS and Tinker DS in 2020, 12% and 28% in 2021, and 14% and 37% in 2022, respectively. By comparison, the relative percentage of the CoR drainage area to the downstream Roanoke River and Tinker Creek stations is 10% and 20% respectively, meaning that the CoR drainage areas had an outsized contribution to streamflow in both CoR Roanoke and CoR Tinker. Although streamflow yield often decreases as watershed area increases, the greater percentages of developed areas and impervious surfaces within the CoR contribution areas likely caused the downstream increase in streamflow yield due to reduced infiltration and increased surface runoff. The CoR’s largest relative contribution to Roanoke DS and Tinker DS occurred during the driest year (2022), suggesting urban streams within the CoR may be a relatively steady source of streamflow during dry years. In fact, streamflow yield at Lick Run, particularly during the lowest and highest observations, was consistently greater compared to the individually monitored watersheds on the Roanoke River or Tinker Creek (Fig. 3 a–c). Developed areas have been shown to have lower evapotranspiration caused by reduced vegetative cover (Bhaskar & Welty, 2012), and previous research in the Eastern United States has indicated that urbanization can cause increased total streamflow and baseflow relative to control watersheds (Bhaskar et al., 2016; Meyer, 2005). Infrastructure leakage and lawn irrigation have been shown to contribute to increased baseflow in some urban streams (Bhaskar et al., 2015; Fillo et al., 2021).

### Suspended-sediment concentrations

Despite greater precipitation and streamflow yield in 2020 compared to 2021 or 2022, the distributions of turbidity among the five monitoring stations were similar between the 3 years (Fig. 3 d–f; Fig. 7 d–f in the Appendix). Unlike streamflow, the turbidity and SSC distributions did not increase from the upstream to downstream monitoring stations on the Roanoke River and Tinker Creek. The lack of downstream increase in turbidity and SSC suggests that sediment and water supply to the channels increase at the same rate, resulting in increased downstream streamflow and SSL but similar SSC. Suspended-sediment may be deposited between sediment-monitoring locations along the Roanoke River which contributed to the lack of downstream increase in turbidity distribution. A majority of the Roanoke River floodplain within the CoR was extensively modified from 2005 to 2012 to reduce the effects of flooding within the city by creating floodplain “benches” (Jastram et al., 2015) which created greater floodplain connectivity and the opportunity for sediment deposition during high streamflow conditions.

**Fig. 4:**
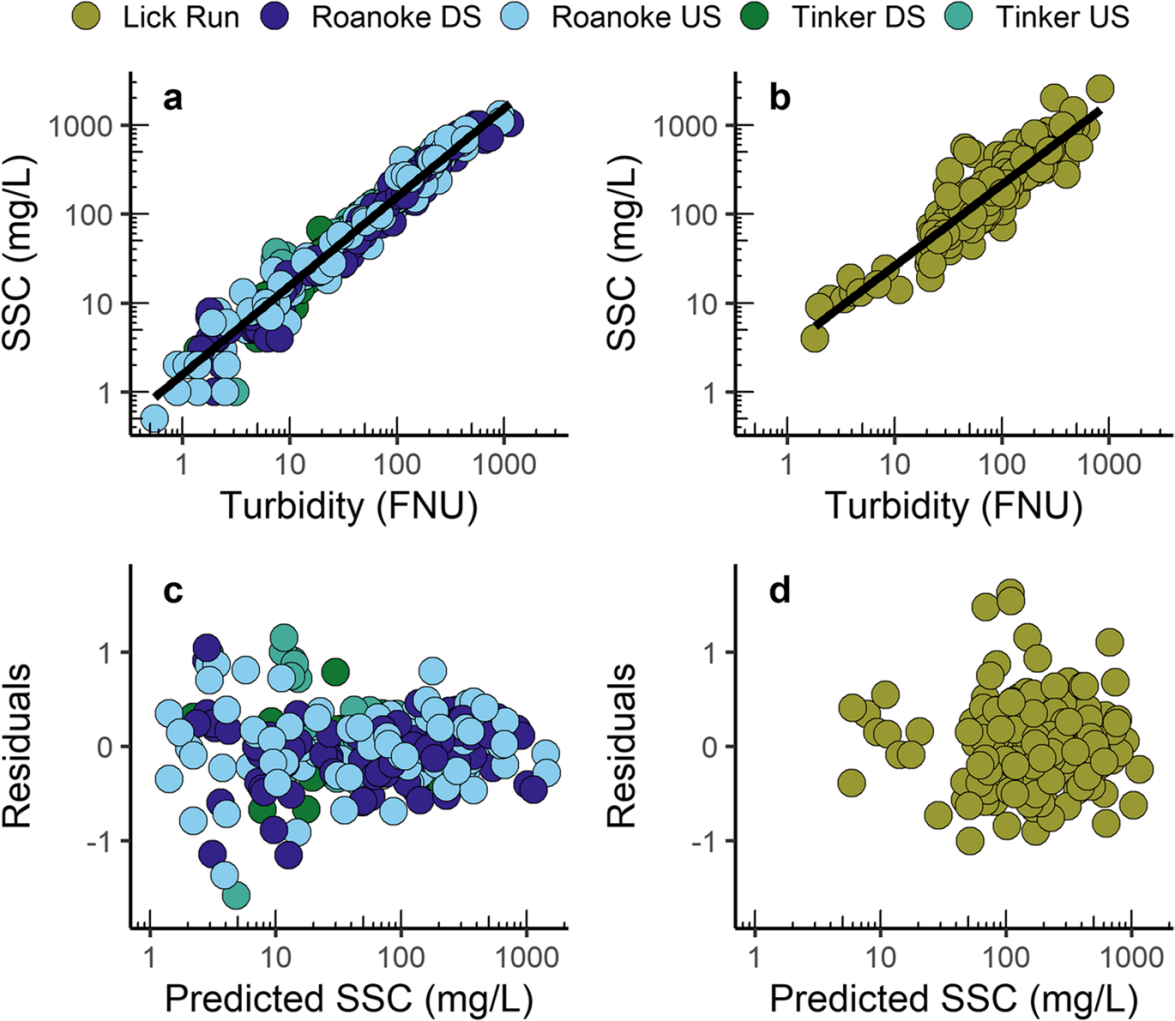
Relation between suspended-sediment concentration (SSC) and turbidity at the Roanoke River and Tinker Creek stations (**a**), and Lick Run (**b**). Residuals versus predicted SSC (**c**–**d**)

Turbidity was a strong predictor of SSC at all sediment-monitoring stations (Fig. 4 a–b). An analysis of covariance revealed no significant differences in the turbidity-SSC relation between the Roanoke River and Tinker Creek sediment-monitoring stations (*p* = 0.017); therefore, a combined model was used to estimate SSC from all samples collected from these four stations. Model uncertainty, indicated by RSE and R^2^_a_, was lowest for the combined Roanoke River and Tinker Creek model, followed by the Lick Run model and models that only used streamflow and seasonality terms (Table 5). All models indicated residual homoscedasticity (Fig. 4 c–d). Surrogate regression models based on only streamflow and seasonality terms were used for the Tinker Creek and Lick Run stations when turbidity data were missing and had greater uncertainty than the turbidity-based surrogate regression models but were used for only 1.0% and 0.4% of observations at Lick Run and the Tinker Creek stations, respectively.

**Table 5:** Model summary diagnostics including the explanatory variables, degrees of freedom (D.F.), residual standard error (RSE), adjusted coefficient of determination (R^2^_a_). For all models, suspended-sediment concentration (mg/L), turbidity (FNU), and streamflow (ft^3^/sec) were natural log-transformed. Regression models with streamflow and seasonality terms are denoted with *

Location	Explanatory variables	D.F.	RSE	R^2^_a_
Roanoke/Tinker	0.45 +1.00(turbidity)	284	0.38	0.95
Lick Run	1.08 + 0.83(turbidity) + 0.11(flow)	178	0.43	0.84
Tinker*	−1.66 + 0.89(streamflow) + 0.42(season)	67	0.79	0.60
Lick Run*	3.17 + 0.64(streamflow) − 0.02(week)	181	0.77	0.52

Turbidity was the only explanatory variable used to predict SSC for the combined Roanoke River and Tinker Creek model (Table 5). In addition to turbidity, streamflow improved the Lick Run model fit and helped remove residual variability. Seasonality terms including the season (meteorological definition) and week in which SSC samples were collected helped remove seasonal effects representing sediment availability that may be caused by seasonal precipitation patterns that can alter turbidity-streamflow relations (Helsel et al., 2020). Similar to turbidity patterns, the distributions of estimated SSC were not substantially different among the 3 years or between upstream and downstream locations on the Roanoke River or Tinker Creek in either year (Fig. 3 e–f; Fig. 7 e–f in the Appendix). Minimum SSC estimated at Lick Run was greater than that at the Roanoke River and Tinker Creek locations. This resulted from elevated SSC measurements at low turbidity and was captured by a different regression model with a greater intercept and different explanatory variables used in the regression model.

### Suspended-sediment loads and yields

**Fig. 5:**
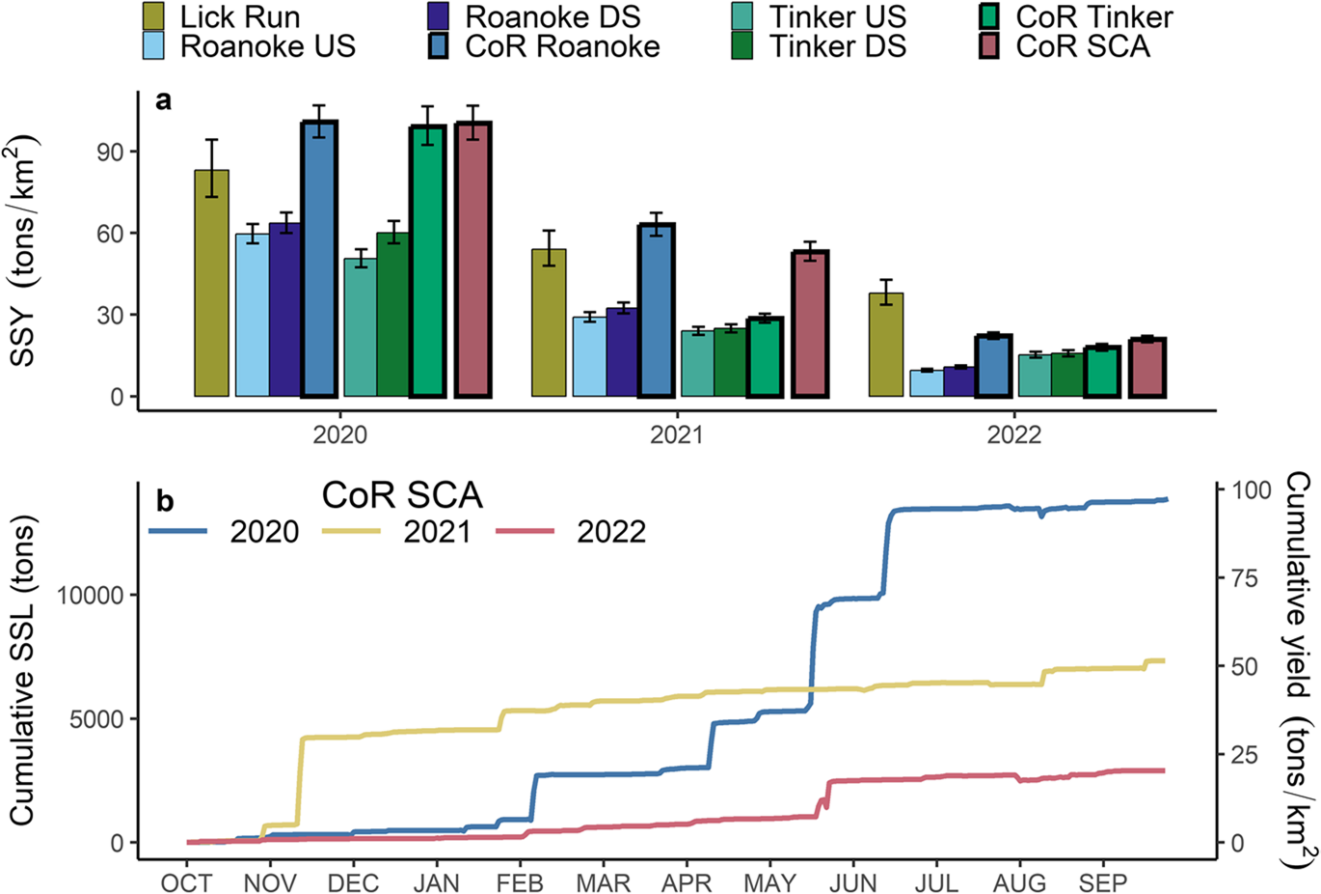
**a** Calculated suspended-sediment yield (SSY) from the five sediment-monitoring stations, contributions from the City of Roanoke to the Roanoke River and Tinker Creek (CoR Roanoke and CoR Tinker), and total contributions from the CoR (CoR SCA). Error bars represent the 95% confidence intervals. **b** Cumulative suspended-sediment load (SSL) and yield (SSY) over time from the CoR SCA during 2020 (blue), 2021 (yellow), and 2022 (red)

Estimated SSLs from all locations in 2020 were about twice those in 2021 and four to six times those in 2022, except for Lick Run, which showed less variability during the 3 years (Table 4). The larger SSLs in 2020 were attributed to greater total precipitation and number of precipitation events (Tables 2, 3). The SSLs generated by CoR Roanoke and CoR Tinker were much smaller than the SSLs generated from areas upstream of the city recorded at Roanoke US and Tinker US. Over the 3-year period, the CoR contributed about 17% of the SSL to Roanoke DS and about 29% of the SSL to Tinker DS. These contributions are higher than would be expected based on land area, as the CoR accounts for only 10% of the Roanoke River and 20% of the Tinker Creek drainage areas. However, the impervious areas within the CoR increase transport capacity and are more susceptible to generating surface runoff with increased velocity and ability to erode streambanks compared to the majority of undeveloped land upstream of the CoR. SSYs tended to be greater in the developed watersheds and contributing areas (Lick Run, Roanoke CoR, Tinker CoR, and CoR SCA), compared to the drainage areas in the Tinker Creek and Roanoke River watersheds upstream of the city which are predominantly forested (Fig. 5a). Downstream SSYs estimated at Roanoke DS and Tinker DS were greater than upstream SSYs at Roanoke US and Tinker US in all years, reflecting the greater SSYs from the more developed and impervious CoR contributing areas.

The SSL estimated from Lick Run, which represents 33% of the CoR Tinker drainage area, accounted for 28%, 62%, and 70% of the SSL from CoR Tinker in 2020, 2021, and 2022, respectively. This implies that Lick Run was a fairly stable source of SSL to CoR Tinker and contributed disproportionally less SSL during the large storm events that were more common in 2020. In fact, Lick Run contributed only about 26% of the streamflow volume and 18% of the SSL to CoR Tinker during the five largest storm events, respectively. Although both Lick Run and CoR Tinker have similar percentages of impervious cover and developed areas, the spatial arrangement of impervious surfaces is likely equally as important for generating large streamflow volumes and SSLs following precipitation events in the area downstream of Lick Run within CoR Tinker. This area drains the central business district in which nearly all streams have been channelized or buried underground and precipitation is routed to the stream channels through storm drains. As a result, the effective imperviousness, the proportion of imperviousness directly connected to streams in CoR Tinker, is likely greater compared to Lick Run, which has been shown to be more strongly correlated with increased bank instability and geomorphic change in urban streams than imperviousness alone (Vietz et al., 2014).

The majority of SSL from the CoR SCA during the 3-year study period occurred during five large storm events (Table 3) and can be seen as abrupt increases in cumulative SSL (Fig. 5b). The mean SSL during these five storms (2952 t—metric tons) was over twenty times greater than the mean from all storms (147 t). Over three-fifths (61%) of the SSL recorded during the 3 years was transported during these five events (14,758 t) but occurred over only 2% of the total time of the study period, similar to results reported by Kemper et al. (2019), who documented 75% of the annual load occurring in about 12 days in Baltimore County, Maryland. Although the average SSY was similar during these five events within CoR Roanoke and CoR Tinker, there was considerable inter-event variability between the two contribution areas which requires further investigation to identify the factors causing the variability in suspended-sediment transport. The large SSLs generated from the five storms were primarily driven by sustained high streamflow and not unusually high SSC, implying the SSL response to the largest precipitation events was sediment-supply limited and driven more by the volume of stream water than the concentration of suspended sediment. For example, smaller precipitation events generated peak turbidity values similar to the five events in Table 3, but peak streamflow and total volume of streamflow were much smaller for other events. In addition, peak streamflow was more strongly correlated to event SSL compared to maximum event turbidity for the downstream Roanoke River and Tinker Creek stations. The 160 storm events identified that produced a single hydrologic response were responsible for 98% of the total CoR SCA SSL, while 2.5-cm storms (*n*=40) were responsible for 86% of the SSL (Table 3). These percentages of total SSL attributed to precipitation events (98%) are much greater than the percentages of total streamflow yield due to precipitation events (64%) and reflect the propensity of suspended-sediment transport during high streamflow and the relative lack of transport during baseflow, a finding documented in previous work (Horowitz, 2009; Vansickle & Beschta, 1983; Wass & Leeks, 1999).

### Evaluation of TMDL target and management implications

The TMDL allocated SSL for the CoR is 1349 t/year; therefore, the TMDL modeled existing SSL that assumes a required 67.5% reduction in SSL to achieve the allocated SSL is 4151 t/year (Table E-6 in Virginia Department of Environmental Quality, 2006). The calculated SSLs in 2020, 2021, and 2022 were 3.4, 1.9, and 0.7 times the TMDL modeled existing SSL and 10.3, 5.8, and 2.1 times the TMDL allocated SSL, respectively. The annual SSY from the CoR SCA during 2020 (100.6 t/km^2^) and 2021 (56.4 t/km^2^) was considerably greater than the TMDL modeled existing SSY (37.4 t/km^2^) or allocated SSY (12.2 t/km^2^); however, during 2022, the SSY (20.8 t/km^2^) was lower than the TMDL-modeled existing SSY, but still greater than the allocated SSY.

There are several reasons that may explain why the calculated SSL from 2020 and 2021 were much greater than the modeled existing SSL in the TMDL including precipitation variability, use of sub-daily SSL estimates, and uncertainty in both the monitored SSL and the TMDL-modeled SSL. Annual precipitation during the TMDL study period (1993–2003) was similar to the long-term average (1913–2022) but much lower than the annual amounts recorded in 2020 and 2021. In fact, the SSY during 2020 and 2021 from the CoR SCA with the five large storm events from Table 3 excluded was less than the modeled existing SSY (37.4 t/km^2^) during 2020 (20.3 t/km^2^) and 2021 (28.5 t/km^2^). Since 1993, annual precipitation in Roanoke, VA, has moderately increased according to the Kendall trend test (tau = 0.22, *p* < 0.1) and climate change predictions suggest there will be increased frequency of large storm events that will increase stormwater runoff from urban areas (Williams et al., 2017). This reiterates the importance of the TMDL being an adaptive process whereby current monitoring data are used to assess progress toward attaining water quality standards, re-evaluate pollutant load goals and management strategies, and adjust the TMDL as needed (United States Government Accountability Office, 2013). Pollutant load reductions designed to achieve TMDL goals may be offset by more frequent, intense storm events in the future; thus, management practices need to be optimized for future climate scenarios (Renkenberger et al., 2017).

The TMDL modeled existing load was based on a daily watershed mass-balance model (Generalized Watershed Loading Functions), which would not capture the sub-daily dynamics of large storm events, particularly in flashy urban streams, to the extent that the 15-min data used in this study did. Since the majority of pollutant loads in urban streams occur during a relatively short period of time, periodic sampling is prone to underestimate pollutant loads (Henjum et al., 2010). A re-calculation of annual CoR SCA SSLs based on turbidity and streamflow daily averages resampled from 15-min data resulted in an 11%, 25%, and 12% decrease in annual SSLs for 2020, 2021, and 2022 (12,550 t, 5892 t, and 2595 t), respectively. Load calculation and modeling contain uncertainty from multiple steps including sample collection, sample analysis, instrument precision, extrapolation, techniques used to estimate fouled data and streamflow, DEM resolution, model input data accuracy, structure, calibration, and validation. It is possible that the monitored and modeled SSL would be more similar if all sources of uncertainty were able to be accounted for.

The importance of storm-event loads in the calculation of annual SSL has previously been noted in this paper, but the importance of time interval in TMDL modeling studies is emphasized here, as the five large storm events (Table 3) each produced an SSL much greater than the annual allocated load for the CoR (1,349 t/year). With respect to the allocated (i.e., “target”) SSL in the TMDL, the allocated SSL for the CoR was modeled based on the percentage reduction needed to achieve the SSL of a relatively undisturbed reference watershed with predominantly forested land cover representing relatively undeveloped conditions. In fact, the TMDL-allocated SSY is near the minimum of SSYs calculated from urban areas and slightly greater than the median SSY from 55 forested watersheds (Russell et al., 2017), which may not be a practical target for highly impervious urban watersheds (Tillinghast et al., 2012). For example, developed urban areas tend to have SSYs roughly six times that of forested watersheds, and three times the SSY of agricultural watersheds (Russell et al., 2017).

The results of this study also provide preliminary insight into implementation of stormwater management practices. Urban stream restoration often focuses on treating the symptoms by modifying and controlling stream channels to accommodate increased stormwater runoff. However, there is little evidence for measurable ecological improvement from such channel reconfiguration restoration projects (Bernhardt & Palmer, 2011; Laub et al., 2012; Violin et al., 2011). Effective management in urban environments likely requires multiple approaches at a range of scales in the watershed that modify the flow regime to reduce the impact on channel morphology (Vietz et al., 2016). Management efforts that do not integrate both the streamflow and sediment regime are unlikely to achieve restoration goals (Wohl et al., 2015). Our results indicate a majority of the annual SSL was transported following a few large precipitation events characterized by exceptional volumes of stream water. Therefore, management strategies that focus on storing water upstream to control the flow regime may have a greater impact on reducing annual SSL compared to strategies solely focused on reducing SSC. However, the volume of storage needed to manage stormwater runoff from these exceptionally large storm events would be similarly large, and the cost and land footprint necessary for this magnitude of storage may be prohibitive.

The CoR has implemented several management practices to comply with the TMDL, including a stream restoration project upstream of the Lick Run monitoring station that began in 2020. Evaluating management practices that have been completed during the period of data collection could provide data to determine the efficacy of the implemented management practices. The goal of the Lick Run restoration project was to reduce sediment loading from instream erosion caused by high volumes and velocity of stormwater runoff by improving floodplain access and re-constituting the riparian buffer, thereby improving instream habitat while increasing park aesthetics. Water-quality monitoring began prior to the construction of this restoration project; thus, data collected at this station present a unique opportunity to analyze the effect of stream restoration on SSL and water-quality response in a small urban watershed. Monitoring at this location could be used to evaluate the effectiveness of the stream restoration project on achieving its initial goals and help the CoR invest in strategies that result in the greatest water-quality improvements.

### Comparison of annual suspended-sediment yields with other studies

The median annual SSY calculated from the developed watersheds and contributing areas in this study (Lick Run, Roanoke CoR, Tinker CoR, and CoR SCA) was 57 t/km^2^ and ranged from 18 to 101 t/km^2^. Jastram et al. (2015) reported a mean SSY estimate for CoR Roanoke from 2006 to 2011 of 23 t/km^2^, which is about one-fifth and one-third of what was calculated during 2020 (101 t/km^2^) and 2021 (63 t/km^2^) for the CoR Roanoke, respectively; however, annual precipitation during that time was generally below average with only 2 years having near-average conditions. In fact, during 2022, which had below average precipitation, annual SSY from CoR Roanoke was 22 t/km^2^.

**Fig. 6:**
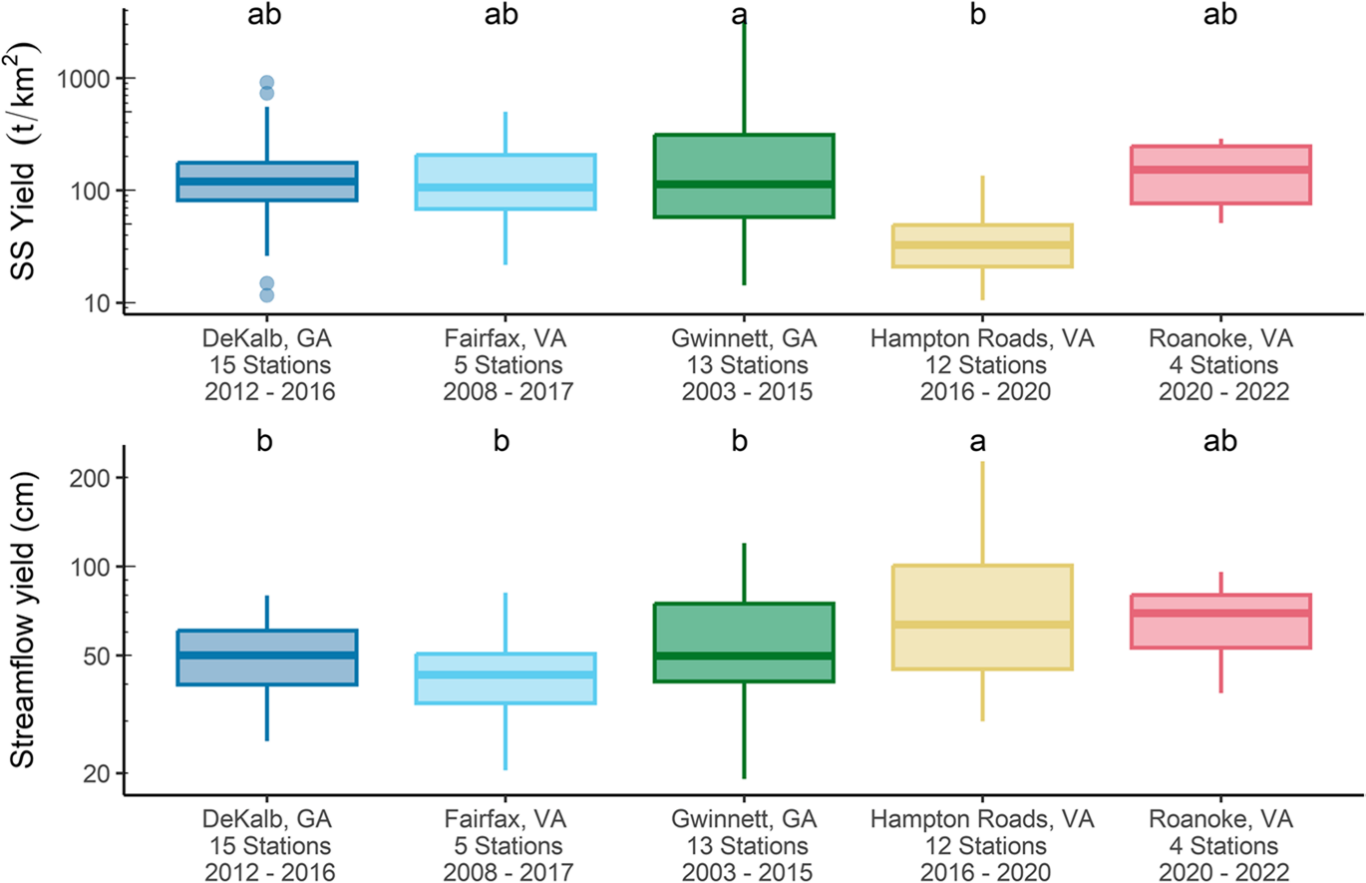
Suspended-sediment yield (top) and streamflow yield (bottom) variability among four USGS studies in urban watersheds (Aulenbach et al., 2017; Aulenbach et al., 2022; Porter, 2022; Porter et al., 2020). Different letters above boxplots denote significant differences according to Tukey’s HSD test at the 0.05 significance level

**Fig. 7:**
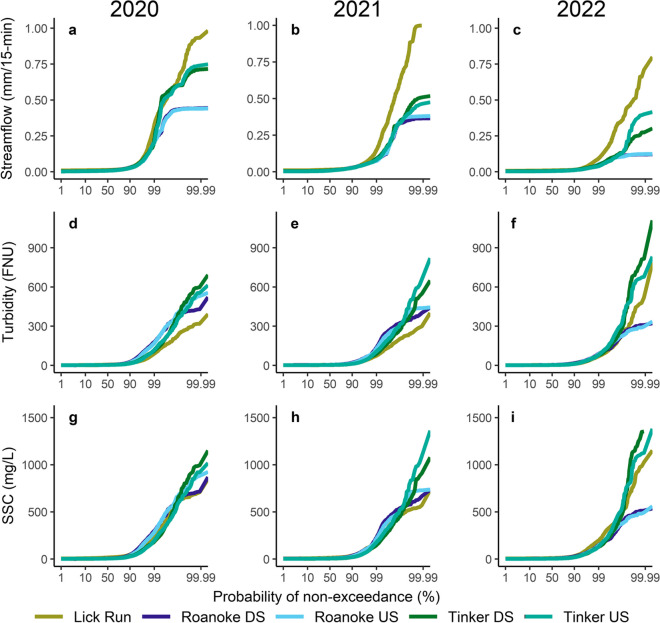
Streamflow yield probability of non-exceedance for the water-quality monitoring stations during 2020, 2021, and 2022 (a, b, c). Turbidity probability of non-exceedance for the water-quality monitoring stations during 2020, 2021, and 2022 (d, e, f). Suspended-sediment concentration (SSC) distributions for all water-quality monitoring stations during 2020, 2021, and 2022 (g, h, i)

Calculated SSYs from this study were similar to those from recent USGS studies from urban and suburban watersheds in the Eastern United States (Fig. 6a) (Aulenbach et al., 2017; Aulenbach et al., 2022; Porter, 2022; Porter et al., 2020). In these studies, SSC was estimated from turbidity surrogate linear-regression models, and the watersheds were generally similar in size and located in nearby regions, and thus have a similar climate. Although the SSYs estimated in this study were similar to those studies, streamflow yield was greater except for the Hampton Roads study (Fig. 6b). Watersheds in the Hampton Roads study were much smaller, had greater percentages of impervious surfaces, and were monitored from stormwater drainage networks which likely led to the greater streamflow yields and comparatively low SSYs due to reduced sediment availability. Variability in SSY was greater in the other studies as they covered a longer period and estimated SSY from more watersheds. In fact, other studies in urban and suburban watersheds in the Eastern United States have estimated similar SSYs with substantial inter-site and interannual variability (Fraley et al., 2009; Gellis et al., 2017; Kemper et al., 2019). The dynamic storage and remobilization of sediment downstream has been hypothesized as a driver of SSY variability in nested urban watersheds whereby upland sediment sources are less frequently mobilized compared to downstream channel deposits that constitute a majority of SSL (Kemper et al., 2019). Additional monitoring in the watersheds analyzed in this study could help to better understand the full range of expected annual SSY under a variety of precipitation conditions.

Regardless of the greatest precipitation recorded in 2020, median annual SSY calculated in this study from the developed watersheds and contributing areas (57 t/km^2^) was substantially lower than the median value reported by Russell et al. (2017) (240 t/km^2^), who summarized SSY from 22 urban watersheds in a global study. However, they also noted large variability, even at a given location, as the 5th and 95th percentile of SSY in their study ranged from 7.1 to 1685 t/km^2^ per year, respectively. Watershed area, slope, climate, annual precipitation, and the extent and organization of urban areas within the watershed influence annual SSY, which may explain the large variability in SSY observed in their global review.

## Conclusions

Excess sediment impacts approximately 224,000 km of rivers and streams in the USA and is a common reason for TMDL development (U.S. Environmental Protection Agency, 2017), yet many TMDL studies lack the high-frequency monitoring data necessary to accurately quantify pollutant loads and assess the impact of management practices on water-quality changes. A novel monitoring design was implemented to estimate the SSL contributed from the CoR to the major waterways intersecting the city. The annual SSL from the CoR to the major waterways draining the city was estimated from 2020 to 2022 using turbidity-SSC surrogate linear-regression models. These 3 years represented above average, average, and below average precipitation for the study area, including the wettest year on record during 2020. Suspended-sediment load during the 3-year period predominately occurred after storm events. Notably, five large precipitation events generated three-fifths of the total SSL. The CoR contributed about 17% of the SSL to the Roanoke River and approximately 29% of the SSL to Tinker Creek. The SSL percentages in these waterways generated by the CoR are greater than the percentages of the watersheds in which the CoR occupies, reflecting greater SSY from parts of these watersheds within the CoR that are more developed and impervious, thus susceptible to increased stormwater runoff, accelerated streambank erosion, and sediment transport capacity. Turbidity and estimated SSC from regression models did not change substantially between the locations where the waterways entered and exited the city boundaries. However, streamflow increases at the downstream stations were apparent, especially during the highest conditions, suggesting that suspended sediment and water supply to the stream channels increased at the same rate within the CoR causing increased downstream streamflow and SSL but similar patterns in SSC.

The SSY from within the CoR was comparable to other monitored urban areas in the Eastern United States; however, the estimated SSL was much greater than the allocated load from the TMDL. The calculated loads during 2 of the 3 years of this study occurred during wetter conditions compared to the time period used for the modeled scenarios from the TMDL, and thus likely reflect the upper estimates for sediment transport. In addition, the modeled TMDL allocated loads utilized daily input data that likely do not capture the dynamics of large storm events, particularly in flashy urban streams, to the extent that the 15-min data used in this study did. SSLs calculated from mean daily data resampled from 15-min data were much lower, but still greater than the allocated SSL. In fact, the mean SSL from the five storm events that generated 61% of the 2020–2022 SSL (2,952 tons) was more than double the TMDL allocated annual SSL (1,349 tons). The TMDL allocated SSY for the CoR is slightly greater than the median SSY from 55 forested watersheds compared in a recent global review. Other TMDL allocated SSLs based on reference watersheds that represent undeveloped areas may not be practical targets for highly impervious urban watersheds. Monitoring suspended sediment under different precipitation scenarios could help inform management efforts that can maximize water-quality improvements.

### Supplementary information

ESM 1 (CSV 159356 kb)

ESM 2 (CSV 74 kb)

ESM 3 (CSV 92894 kb)

ESM 4 (CSV 2 kb)

## Data Availability

The authors confirm that the data supporting the findings of this study are available within the article or its supplementary materials.

## Appendix

**Table 6:** First and second dominant and secondary lithologies as a percentage of drainage area for the study watersheds and contributing areas (Dicken et al., 2005)

	1st dominant lithology		2nd dominant lithology		1st secondary lithology		2nd secondary lithology	
Name	Type	(%)	Type	(%)	Type	(%)	Type	(%)
Lick Run	Shale	51	Dolostone	49	Limestone	49	Siltstone	35
Roanoke US	Shale	42	Dolostone	15	Siltstone	27	Limestone	20
Roanoke DS	Shale	45	Dolostone	15	Siltstone	31	Limestone	19
Tinker US	Shale	34	Dolostone	31	Limestone	33	Mudstone	19
Tinker DS	Shale	38	Dolostone	32	Limestone	33	Siltstone	18
CoR Roanoke	Shale	77	Dolostone	10	Siltstone	66	Limestone	10
CoR Tinker	Shale	56	Dolostone	36	Siltstone	51	Limestone	36
CoR SCA	Shale	71	Dolostone	17	Siltstone	62	Limestone	18
CoR Admin	Shale	70	Dolostone	24	Siltstone	68	Limestone	24

**Table 7:** Hydrologic soil group as a percentage of drainage area for the study watersheds and contributing areas (Soil Survey Staff, 2020). Runoff potential increases from soil group A to soil group D

	National Resource Conservation Service Hydrologic Soil Group (%)				
Name	A	B	C	D	Uncategorized
Lick Run	1	31	8	17	44
Roanoke US	21	44	20	14	1
Roanoke DS	20	42	18	17	2
Tinker US	12	46	18	17	7
Tinker DS	9	44	15	19	12
CoR Roanoke	16	26	4	44	11
CoR Tinker	1	36	3	28	31
CoR SCA	11	29	4	39	17
CoR Admin	10	28	2	38	22
